# Anal Injury in a Neonate due to Artificial Rupture of Membrane

**Published:** 2014-07-10

**Authors:** Mamatha Basavaraju, Dhiraj K Balaji

**Affiliations:** Department of Pediatric surgery, Vydehi Institute of Medical Science and Research Centre Whitefield, Bangalore -66

**Dear Sir**

A 26-year-old primigravida with breech presentation came to the emergency in 2nd stage of labor with meconium stained per vaginal leak. Patient was initially taken to a primary health centre were artificial rupture of membrane was done to augment labor but later referred here since labor did not progress. After an emergency cesarean section, a female baby was delivered who on examination had a hematoma over right buttock, anal laceration, perianal edema, and severe rectal bleeding (Fig. 1). Anal injury during artificial rupture of membrane was suspected. Patient was managed conservatively by anal pack for 24 hours and later local dressings and antibiotics. The edema and hematoma resolved in 1 week (Fig. 2). The child has normal continence. 

Intrauterine anogenital injury can occur due to repeated maternal vaginal examination in which the examiners fingers traverses the cervical os into the neonatal rectum or vagina. It can also occur during cesarean delivery due to blunt dissection of endometrium or bimanual traction while delivering the breech from maternal pelvis. There are very few case reports regarding anal injuries in breech delivery, [1-4] but this is probably the first such incidence where it occurred due to artificial rupture of membranes. This injury stimulated baby to pass meconium in-utero which could be life threatening. Mal-presentation is a contraindication for labor augmentation or induction procedures like artificial rupture of membrane. Management of such injuries depends on the grade of injury.[5] Mild degree laceration can be managed conservatively like in our case. The more severe degree laceration of sphincter or those extending into rectum or genitalia are managed by a diverting stoma and primary repair. [1-4]

**Figure:**
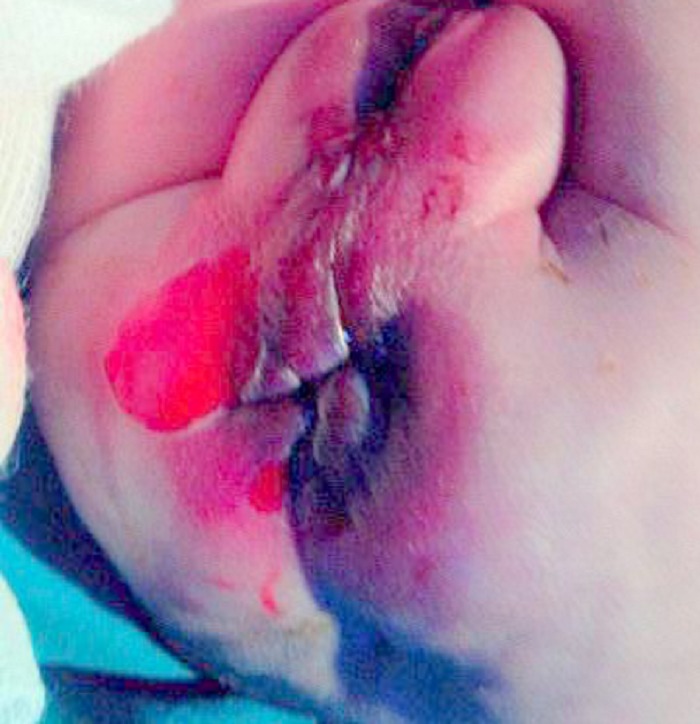
Figure 1: Hematoma over right buttock 4x4cm with abrasion of overlying skin, anal laceration of 0.5 x 1cm, and perianal edema.

**Figure:**
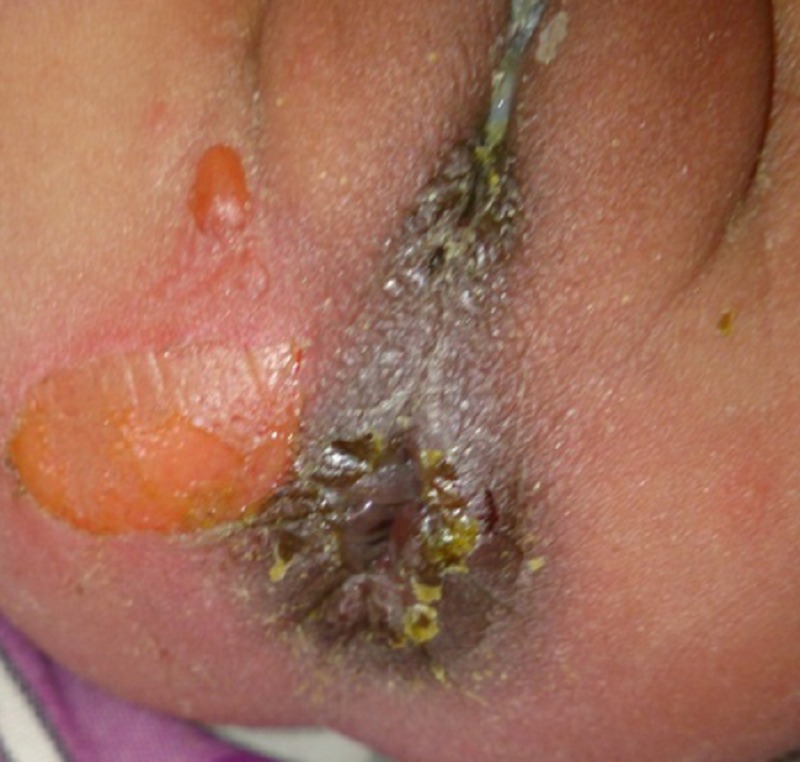
Figure 2: Resolution of edema and hematoma with progressing healing after a week.

## Footnotes

**Source of Support:** Nil

**Conflict of Interest:** None

